# Refining national greenhouse gas inventories

**DOI:** 10.1007/s13280-019-01312-9

**Published:** 2020-01-24

**Authors:** Leehi Yona, Benjamin Cashore, Robert B. Jackson, Jean Ometto, Mark A. Bradford

**Affiliations:** 1 School of Forestry and Environmental Studies, Yale University, 195 Prospect Street, New Haven, CT 06511 USA; 2 Emmett Interdisciplinary Program in Environment and Resources, Stanford University, 473 Via Ortega, Suite 226, Yang and Yamazaki Environment and Energy Building, Stanford, CA 94305 USA; 3 Lee Kuan Yew School of Public Policy, National University of Singapore, Singapore, 259772 Singapore; 4 Department of Earth System Science, Woods Institute for the Environment, and Precourt Institute for Energy, Stanford University, 473 Via Ortega, Room 140, Stanford, CA 94305 USA; 5 Center for Earth Systems Science, National Institute for Space Research, CCST/INPEAv dos Astronautas, 1.758, São José dos Campos, SP CEP 12227-010 Brazil

**Keywords:** Climate change, Forest soil carbon, Global carbon cycle, Greenhouse gas emissions inventories, Intergovernmental Panel on Climate Change, United Nations Paris Agreement

## Abstract

The importance of greenhouse gas inventories cannot be overstated: the process of producing inventories informs strategies that governments will use to meet emissions reduction targets. The Intergovernmental Panel on Climate Change (IPCC) leads an effort to develop and refine internationally agreed upon methodologies for calculating and reporting greenhouse gas emissions and removals. We argue that these guidelines are not equipped to handle the task of developing national greenhouse gas inventories for most countries. Inventory guidelines are vital to implementing climate action, and we highlight opportunities to improve their timeliness and accuracy. Such reforms should provide the means to better understand and advance the progress countries are making toward their Paris commitments. Now is the time to consider challenges posed by the current process to develop the guidelines, and to avail the policy community of recent major advances in quantitative and expert synthesis to overhaul the process and thereby better equip multi-national efforts to limit climate change.

## Introduction

The importance of greenhouse gas (GHG) inventories cannot be overstated: the process of producing inventories informs and influences strategies that governments might use to meet emissions reduction targets. Further, both international policy negotiations and domestic policy interventions designed to achieve climate action depend on accurate monitoring and reporting of emissions. To this end, the Intergovernmental Panel on Climate Change (IPCC), best known for its Assessment Reports on human-induced climate change (Carraro et al. 2015; Hallegatte and Mach 2016; IPCC 2006, 2014), also leads a second effort to develop internationally agreed upon guidelines for calculating and supporting national reports on GHG emissions and removals. These IPCC national GHG inventory guidelines (henceforth “IPCC guidelines”) play an important role in fostering the incorporation of scientific evidence into national climate policy mechanisms. They are of particular interest now, as they were just “refined” in 2019, the first major overhaul since 2006 (Jamsranjav 2017).

The IPCC guidelines to calculate national GHG inventories are widely applied by signatories to the United Nations Framework Convention on Climate Change (UNFCCC). The UNFCCC has historically adopted various versions of these IPCC guidelines for their own reporting purposes (and we refer to guidelines in this context as “UNFCCC guidelines”). Currently, the 2006 IPCC guidelines are used by Annex I countries; non-Annex I countries are still in the process of developing GHG emissions inventories, and typically refer to the 1996 IPCC guidelines, as they are not required to use the 2006 version. Establishing accurate inventories is essential for developing meaningful national-level mitigation commitments, which form the bedrock of the Paris Agreement (Jamsranjav 2017).

The IPCC guidelines themselves are produced using established procedures involving authors, a large number of co-authors, and expert and governmental review. These are the same procedures used to produce the Assessment Reports. However, the IPCC guidelines receive far less media reporting, public interest, and academic attention than the Assessment Reports. This relative obscurity is challenging for a number of reasons because, among other factors, it makes it difficult to evaluate both the degree to which the self-interests of nation states shape the IPCC guidelines and the extent of data available for any one country to inform emissions estimates. In contrast, the Assessment Reports are considered to be based on the best available science, independent of national interests, and hence serve as de facto reference documents for policymakers.

Here, we argue that national GHG inventory guidelines are vital to climate action and highlight important opportunities to improve their timeliness and accuracy. Such reforms should provide the means to both better understand and further the progress countries are making toward their Paris commitments. In this regard, two related activities serve as examples of the kinds of opportunities available to modify the approach to developing the IPCC guidelines to better reflect the advances in scientific knowledge required for countries to achieve their Paris commitments. The first activity is the 2019 Refinement—the IPCC effort to provide updates to existing inventory guidelines—completed in May 2019. The second activity is a set of processes, the Talanoa Dialogue and Global Stocktake, which involve efforts to help take stock of each country’s progress in meeting their GHG emissions reduction commitments. We argue that both sets of activities present opportunities to incorporate advances in scientific synthesis to make the IPCC guidelines more effective. We first lay out challenges about the current process to produce the IPCC guidelines. We then describe the recommendations to incorporate these advances.

## Discussion

### Challenges for IPCC guidelines: Writing process

The process by which the IPCC guidelines are developed and updated can be improved. There are opportunities to bolster the way the current guidelines handle the complexities of GHG reporting for all Paris Agreement countries. In particular, the IPCC guideline development process has not been modified since its inception, which was introduced in recognition of the challenge of synthesizing large amounts of information. In particular, the process uses expert synthesis instead of structured, quantitative synthesis; hence the “views” represented in the guidelines are strongly susceptible to individual interpretation. Even in recent guideline updates (e.g., the 2019 Refinement), the IPCC continues to use the same expert-synthesis process first used in 1996 (Jamsranjav 2017). Yet, along with the increasing availability of large-scale datasets, the capacity to synthesize scientific information has substantively increased over the last two decades; new approaches exist to update the process with state-of-the-art of synthesis approaches.

The nearly quarter century-old expert-synthesis process, currently used by the IPCC, involves four stages for developing guidelines, whereby government-nominated experts write and revise drafts. Following a first drafting process in which scientists collectively agree, a second draft (referred to as “First Order”) is available for comment by additional self-nominated expert reviewers. The third (“Second Order”) and fourth (“Government”) drafts are then made available to the IPCC’s government members for comment, with expert reviewers commenting on the third draft as well. The last step in this expert-synthesis process involves final plenary approval in the IPCC session by member states.

The rationale for this four-stage process is to foster careful deliberations about how to measure and report GHG emissions. However, the IPCC guideline production process can sometimes impede the diffusion of new scientific research once the process is initiated, owing in part to publication deadlines for including peer-reviewed research in the writing process. Hence, we see an opportunity to reform the IPCC guideline process to foster greater accuracy and timeliness for the science of GHG emissions and reporting, as described below under “Recommendations”.

### Challenges for IPCC guidelines: Reporting methodologies

A second challenge lies within the methodology for reporting GHG inventories. To develop inventories, nation states apply what are known as Tier 1, 2, and 3 methodologies. Tier 2 and 3 methodologies generate emissions and uptake estimates with a relatively high degree of spatial and contextual resolution (IPCC). Tier 1 methods, on the other hand, provide default methods and values that have less resolution compared to Tier 2 and 3 methodologies (Intergovernmental Panel on Climate Change). Under the Kyoto Protocol, Annex I countries were expected to mostly apply Tier 2 or 3 approaches when monitoring their commitments. Not only were they the only countries that committed to substantive binding reductions but they also had the greatest capacity and expertise to undertake Tier 2 and 3 methodologies. However, these approaches pose challenges to the implementation of the Paris Agreement, since the participation of non-Annex I countries means that Tier 1 methodologies may become the default approach, rather than Tier 2 or 3 under Kyoto. A challenge arises wherein Tier 1 methods may not incorporate nuanced science about complex sinks and sources at the sectoral and geographic scales at which actions are carried out, leading to poor quantification of the effectiveness of GHG emissions-based interventions.

However, countries are encouraged to use Tier 2 or 3 methods, particularly under Article 13 of the Paris Agreement, but many do not have the scientific resources and capacity to do so. Additionally, although the IPCC encourages use of its Emission Factor Database by countries to find up-to-date information, this effort is under-resourced; the emissions values are drawn from the IPCC guidelines themselves. Importantly, to our knowledge there are no mechanisms within the current process of producing the guidelines to incorporate emerging scientific knowledge into Tier 1 methodologies; once Tier 1 guidelines are set, it is not easy to change them. We acknowledge that many Annex I countries rely primarily on Tier 2 and 3 approaches but at the same time use them for some sectors. At the same time, Tier 1 inventory approaches are relied on primarily by non-Annex I countries, who are responsible for a sizable portion of global emissions. As such, it is worth considering how these IPCC processes, such as the guidelines and Emission Factor Database, might be improved to facilitate their use for Tier 1 inventory compiling.

The UNFCCC Subsidiary Body for Scientific and Technological Advice (SBSTA) has not yet adopted the 2019 Refinement as part of the UNFCCC guidelines, but will likely consider it at an upcoming meeting. Assuming the Refinement is adopted, all Paris Agreement signatory nations—who will become mandatory inventory compilers beginning in 2020—will be making use of IPCC inventory guidelines. Because many signatory parties lack the data and scientific infrastructure for Tier 2 and 3 methodologies, Tier 1 methods are poised to take an even more central role in GHG inventories and follow-up commitments. Given the existing expert-synthesis process used to refine the IPCC guidelines, nations using Tier 1 approaches will therefore not be able to take advantage of emerging scientific information that might substantively alter their quantification of emissions and removals, and subsequently might impact policy decisions.

The UNFCCC decision about adoption is significant for assessing and understanding what GHG emissions reductions policies countries undertake. For example, within the forestry sector, Tier 1 methods provide default values for soil carbon stocks in forests, ecosystems that store more carbon than is contained in the atmosphere (Trumbore 2009). Under Tier 1 guidelines, conversion of primary forest to secondary or plantation forest assumes no losses of mineral soil organic carbon (IPCC). This assumption contrasts with results of large data syntheses that suggest substantive losses due to conversion in many instances, particularly to prominent plantation trees such as *Pinus* (Berthrong et al. 2009; Liao et al. 2010). Countries with Tier 2 and 3 inventory capabilities can avail of such new information to inform their compilations, but countries relying on Tier 1 are unable to do so, because they lack primary data. Selective harvesting is another practice where recent research has revealed greater negative impacts on forest carbon stocks than previously assumed (Bustamante et al. 2016). Such forest degradation affects ~ 100 million hectares annually, is difficult to detect without remote sensing technologies, and is included primarily through the category of “managed lands” (Lewis et al. 2015). Notably, this new scientific information on forest soil organic carbon stocks—as is typical also for many other new research insights—was not thoroughly updated in the 2019 Refinement (IPCC 2016, 2019). Hence, even with the Refinement, many nations will be obliged to use outdated scientific information for inventory compiling if Tier 1 guidelines continue to be revised using expert synthesis alone.

Of particular concern, therefore, is the apparent lack of a mechanism to use emerging scientific information to modify Tier 1 assumptions and default emissions values in real time after a refinement process. Tier 1 methods for GHG inventories age rapidly even when they reflect the best contemporary knowledge at the time of incorporation. We can expect the rate at which they become dated only to accelerate given the era of big data science, where the capacity to measure and synthesize data for carbon stocks and GHG emissions is rapidly expanding. This reality poses challenges to the UNFCCC’s principles of transparency, accuracy, completeness, comparability, and consistency (TACCC) in its efforts to meet Paris Agreement targets.

### Recommendation: Use increased technological capacities

Here, we suggest actions for ensuring that Tier 1 approaches are based on the best available science. First, technological innovation in the availability and evaluation of literature—including machine learning tools to expedite qualitative and quantitative synthesis—opens doors to reconceive guideline development. One issue is that guideline development is systematically under-resourced both financially and in human capacity. We can, however, help address scientific knowledge gaps by making available resources to avail of big data—such as the ever-increasing availability of research in remote sensing and machine learning—while also being mindful of countries’ national-level resource constraints. For example, in the case of emissions and sinks related to land use, remote sensing and machine learning approaches are increasing the accuracy of carbon accounting (Gibril et al. 2018; Aburas et al. 2019; Chen et al. 2019; Delalay et al. 2019; Sharififar et al. 2019). The approaches, combined with data products such as NASA satellite imagery and the Global Environmental Outlook datasets (United Nations System-Wide EARTHWATCH > Data & Observation 2019), could substantively assist countries lacking primary national-level data on land use and management. Such interventions could include adopting a systematic review process that combines scientific knowledge about carbon emissions and sinks with land cover data to compile inventories. Applying such technologies will require careful planning to avoid unintended challenges, such as technocratic environmental solutions, the ethics of environmental monitoring (Bakker and Ritts 2018), or an over-emphasis on quantitative values (Wasserstein and Lazar 2016).

### Recommendation: Use a Cochrane Collaboration inspired approach

Second, we suggest that recent innovations offer promise in addressing the challenges of accurate GHG reporting, consistent with the Paris Agreement’s commitment to developing a “rigorous” transparency framework for understanding how signatory countries develop their domestic GHG inventories. Such an impetus would stimulate science and policy processes to be more mutually informative. Developing standard reporting in line with IPCC methods would remove an important impediment to the production of guidelines that reflect the most up-to-date literature, and therefore help to generate science that both informs, and is informed by, the policymaking process. We recognize both resource-based and political challenges to implementing these changes and do not want to downplay these potential barriers, especially for countries relying primarily on Tier 1 approaches. Nonetheless, we hope that the provisions of Article 13 of the Paris Agreement pertinent to reporting—provisions for supporting countries to meet reporting requirements, as well as technology transfer and capacity-building for transparency—can help alleviate some of these resource challenges. With this assumption in mind, we propose a dynamic empirical review approach to more accurate GHG emissions reporting, and thus more effective policymaking for climate action. Such an approach should explicitly integrate science with policy for environmental stewardship (Yona et al. 2019).

A dynamic review approach could be modeled on the Cochrane Collaboration used in medicine and health science (Weeks 2013). The Collaboration informs policy and practice interventions through synthesis of the latest scientific knowledge, including peer-reviewed data and any pre-publication breakthroughs that scientists consider sufficiently advanced and rigorous to merit consideration. Such developments reflect the spirit of the Cochrane Collaboration, which emphasizes data that are both accessible and relevant to researchers and the public. In fact, the Collaboration goes well beyond preprints, encouraging contributors to provide new datasets for quantitative syntheses, thus ensuring the best available data are used to inform human-health interventions. The Cochrane Collaboration therefore could help make IPCC guidelines more accurate by better representing the most up-to-date, peer-reviewed scientific evidence.

Logistically, each Cochrane Review Group undertakes a systematic review related to a specific issue and is housed at a research institution. A similar approach could be used for the IPCC’s different GHG sectors, with the recent proliferation of environmental synthesis centers, such as the National Center for Ecological Analysis and Synthesis (Environmental Data Science | NCEAS 2019), already providing potential venues for such efforts. Similarly to the current Cochrane approach, various research institutions may target a different sector of the global GHG budget (for example, the electricity sector, or forestry management), consistent with a “wedges” approach (Pacala and Socolow 2004). These centers would then, in a Cochrane approach, conduct systematic reviews in their sectors, contributing to GHG inventory guidelines. The Cochrane Collaboration has been beneficial to the medical field in that it has helped synthesize large datasets on a timeline more consistent with the policymaking process and with implementation into practice. We expect a similar approach for GHG inventories would produce guidelines grounded more fully in up-to-date empirical data and understanding, which should lead to more robust inventories and climate policies. The most recent science needs to be reflected in policy recommendations, and inclusiveness (e.g., broad scientific community representation) based on best available science is critical for success.

### New opportunities for greenhouse gas inventories

Greenhouse gas inventory guideline development is a pressing issue. If ever-increasing climate mitigation efforts are to succeed, they must be informed using a dynamic scientific approach to measure GHG emissions. The IPCC’s GHG inventory guidelines are fundamental to climate change mitigation and a powerful tool to realize mitigation goals. To realize this potential, the guideline process should use the most modern approaches for scientific synthesis, and should update its methodologies closer to real time as new information becomes available. Our two recommendations are based on major advances in quantitative and expert synthesis that we believe the guideline production process should employ to ensure guidelines are based on the best available science. With advances in technological analysis and big data, we can substantively improve how we measure, report, and account for GHG emissions. A further opportunity exists for real-time incorporation of insights from new data through formal collaborative synthesis with expert groups in different GHG sectors. The IPCC’s guidelines for GHG inventories form an important foundation for climate policy. Now is the time to improve the reliability and accuracy of emissions and stock inventories to achieve climate mitigation goals.
